# Virtual reality-based treatment for regaining upper extremity function induces cortex grey matter changes in persons with acquired brain injury

**DOI:** 10.1186/s12984-020-00754-7

**Published:** 2020-09-12

**Authors:** Jiří Keller, Ivana Štětkářová, Vince Macri, Simone Kühn, Jakub Pětioký, Stefano Gualeni, С. Douglas Simmons, Sajay Arthanat, Paul Zilber

**Affiliations:** 1 Department of Radiology, Na Homolce Hospital, Prague, Czech Republic; 2 Department of Neurology, Third Faculty of Medicine, Charles University, Prague, Czech Republic; 3 3D PreMotorSkill Technologies LLC, Tallahassee, FL USA; 4 Department of Psychiatry and Psychotherapy, University Clinic Hamburg-Eppendorf, Hamburg, Germany; 5 REGIBASE, Prague, Czech Republic; 6 Rehabilitation Center, Kladruby, Czech Republic; 7 Institute of Digital Games, University of Malta, Msida, Malta; 8 School of Occupational Therapy, MCPHS University, Manchester, NH USA; 9 Department of Occupational Therapy, University of New Hampshire, Durham, NH USA

**Keywords:** Acquired brain injury (ABI), Virtual anatomical interactivity (VAI), Brain plasticity, Magnetic resonance imaging (MRI), Rehabilitation, Stroke, Traumatic brain injury (TBI), Virtual world, Therapeutic games, Video-observation-feedback therapy

## Abstract

**Background:**

Individuals with acquired brain injuries (ABI) are in need of neurorehabilitation and neurorepair. Virtual anatomical interactivity (VAI) presents a digital game-like format in which ABI survivors with upper limb paresis use an unaffected limb to control a standard input device and a commonplace computer mouse to control virtual limb movements and tasks in a virtual world.

**Methods:**

In a prospective cohort study, 35 ambulatory survivors of ABI (25/71% stroke, 10/29% traumatic brain injury) were enrolled. The subjects were divided into three groups: group A received VAI therapy only, group B received VAI and physical/occupational therapy (P/OT), and group C received P/OT only. Motor skills were evaluated by muscle strength (hand key pinch strength, grasp, and three-jaw chuck pinch) and active range of motion (AROM) of the shoulder, elbow, and wrist. Changes were analyzed by ANOVA, ANCOVA, and one-tailed Pearson correlation analysis. MRI data was acquired for group A, and volumetric changes in grey matter were analyzed using voxel-based morphometry (VBM) and correlated with quantified motor skills.

**Results:**

AROM of the shoulder, elbow, and wrist improved in all three groups. VBM revealed grey matter increases in five brain areas: the tail of the hippocampus, the left caudate, the rostral cingulate zone, the depth of the central sulcus, and the visual cortex. A positive correlation between the grey matter volumes in three cortical regions (motor and premotor and supplementary motor areas) and motor test results (power and AROM) was detected.

**Conclusions:**

Our findings suggest that the VAI rehabilitation program significantly improved motor function and skills in the affected upper extremities of subjects with acquired brain injuries. Significant increases in grey matter volume in the motor and premotor regions of affected hemisphere and correlations of motor skills and volume in nonaffected brain regions were present, suggesting marked changes in structural brain plasticity.

**Trial registration:**

The trial “Limitations of motor brain activity – use of virtual reality for simulation of therapeutic interventions” has been registered under reference number ISRCTN11757651.

## Background

Neurological disorders, including acquired brain injuries (ABIs) are important causes of disability and death worldwide [1, 2]. Although age-standardized mortality rates for ischemic and hemorrhagic strokes have decreased in the past two decades, the absolute number of stroke survivors is increasing, with most of the burden in low- and middle-income countries [3]. Another major issue is that trends toward increasing stroke incidence at younger ages has been observed [4]. Moreover, this type of ABI is the leading cause of long-term disability in the United States, with an estimated incidence of 795,000 strokes yearly [2].

In more than 80% of stroke survivors, impairments are seen in at least one of the upper limbs. Six months after a stroke, 38% of patients recover some dexterity in the paretic arm, though only 12% recover substantial function even in spite of having received physical/occupational therapy (P/OT) [5]. Only a few survivors are able to regain some useful function of the upper limb. Failing to achieve useful function has highly negative impacts on the performance of daily living activities [6, 7]. Regaining control and improving upper limb motor function after ABIs are therefore crucial goals of motor system rehabilitation. In left-sided limb impairment, neglect syndrome can contribute to a worsened clinical state, making the alleviation of symptoms even more difficult to achieve. Mirror therapy has been reported as a promising approach to improve neglect symptoms [8, 9].

MRI has been used to track changes in brain connectivity related to rehabilitation [10], and several studies of healthy individuals playing off-the-shelf video games have demonstrated changes in the human brain resulting from interactions in a virtual world (VW) [11, 12]. Furthermore, playing video games results in brain changes associated with regaining improved, purposeful physical movements [13, 14]. The socio-cultural relevance of virtual reality (VR) and VW applications lies, more generally, in the fact that these technologies offer interactive environments to users. These interactive environments are actually present in the users’ experiences while less so in the world they share as biological creatures [15]. The way in which we engage with VWs allows for rehabilitation exercises and activities that feel similar to their actual physical world counterparts [11]. In the past two decades, researchers have demonstrated the potential for the interactive experiences of VWs to provide engaging, motivating, less physically demanding, and effective environments for ABI rehabilitation [9, 16–18].

One of the suitable rehabilitation methods seems to be exercises and tasks in VW called virtual anatomical interactivity (VAI) [19]. This method provides sensory stimulation / afferent feedback and allows the independent control of an anatomically realistic virtual upper extremity capable of simulating human movements with a true range of motion. ABI survivors are able to relearn purposeful physical movements and regain movement in their disabled upper extremities [19]. Contrary to conventional therapy, which exercises impaired upper limbs to improve limb movement, the general VAI hypothesis is that brain exercises alone (or combined with traditional therapy) may positively influence neuroplastic functions. In the VW, subjects can move their virtual impaired limbs using their healthy hands, meaning simulated physical movements are survivor-authored. Virtual visuomotor feedback may help regain functional connectivity between the brain and the impaired limb, therefore also regaining voluntary control of the limb.

The aim of the study was to test if the shoulder, elbow, and wrist movement; hand pinch strength; and grip strength of the paretic side improved through the use of VAI exclusively or combined with P/OT for upper extremities and how these approaches improved functional outcomes measured by the Action Reach Arm Test [20]. The relationship between changes in abilities to control upper extremities and volumetric changes in cortex grey matter measured by VBM and using MRI was also explored.

## Methods

There were 35 subjects in this 10-week study (25 stroke, 10 TBI; 20 male, 15 female; average age: 46 years; age range: 26–72; average elapsed time postinjury: 3.6 years) divided into three groups (summarized in Tables 1 and 2): group A received VAI only (with no P/OT), group B received both VAI and P/OT, and group C with P/OT only.

**Table 1:** Group characteristics and exercise duration. S = stroke, TBI = traumatic brain injury. SD = standard deviation of hours of intervention

Group	Male/ Female	S/TBI	Average age	Average years after injury	Average hours of intervention (SD)
**Group A** (VAI Only)	9/5	11/3	50.50	6.93	14.4 (5.4)
**Group B** (VAI and P/OT)	5/8	10/3	46.85	1.62	264.5 (78.9)
**Group C** (P/OT Only)	6/2	4/4	37.50	1.13	211.0 (54.6)
**All**	20/15	25/10	45.45	3.6	152.2 (127.6)

**Table 2:** Demographic information on patients. ID = group and patient ID, M/F = gender (M = male, F = female), Age = age at onset of rehabilitation, Dg. = diagnosis (R = right-sided, L = left-sided, S-R = stroke in the right hemisphere, S-L stroke in the left hemisphere, TBI = traumatic brain injury caused by motor vehicle accident), YPI = years postinjury, IE = impaired upper extremity (R = right, L = left). Patients from group A who underwent MRI are in **bold**, patients who did not (due to contraindication of MRI) are in *italics*

ID	M/F	Age	Dg.	YPI	IE	ID	M/F	Age	Dg.	YPI	IE	ID	M/F	Age	Dg.	YPI	IE
**A01**	**M**	**39**	**S-R**	**4**	**L**	B01	F	59	S-R	1	L	C01	F	37	S-L	1	R
**A02**	**M**	**65**	**S-R**	**5**	**L**	B03	F	23	TBI	1	R	C02	M	29	TBI	1	L
*A04*	*M*	*29*	*TBI*	*10*	*R*	B04	F	56	S-L	1	R	C03	M	42	S-L	2	R
**A05**	**M**	**65**	**S-L**	**4**	**R**	B06	M	59	S-L	1	R	C04	M	54	S-L	1	R
**A06**	**M**	**63**	**S-L**	**1**	**R**	B07	F	60	S-R	9	L	C05	M	60	S-R	1	L
**A07**	**M**	**62**	**S-R**	**6**	**L**	B10	F	36	S-R	1	L	C07	M	35	TBI	1	L
*A08*	*F*	*52*	*S-L*	*5*	*R*	B12	M	53	S-L	1	R	C11	M	21	TBI	1	L
*A09*	*M*	*72*	*S-L*	*3*	*R*	B15	F	30	TBI	1	L	C12	F	22	TBI	1	L
**A10**	**F**	**64**	**S-L**	**5**	**R**	B17	M	42	TBI	1	L						
**A11**	**F**	**29**	**S-R**	**6**	**L**	B19	F	43	S-L	1	R						
*A12*	*M*	*44*	*S-L*	*26*	*R*	B20	F	54	S-L	1	R						
**A13**	**M**	**62**	**S-R**	**12**	**L**	B21	M	51	S-R	1	L						
**A14**	**F**	**35**	**TBI**	**9**	**L**	B23	M	43	S-L	1	R						
**A15**	**F**	**26**	**TBI**	**1**	**L**												

The inclusion criterion was isolated motor impairment of one upper extremity either from MCA stroke or trauma; the exclusion criterion was the cognitive or physical inability to control a standard computer mouse.

MRI was performed for a subgroup of 10 subjects from group A (several individuals had metal implants, one had claustrophobia), pre- and postintervention. Detailed subject characteristics, including clinical status, diagnosis, age, gender, and duration from ABI onset are summarized in Table 2. Rehabilitation took place at inpatient and outpatient facilities: inpatient at the Kladruby Rehabilitation Center, where subjects were randomized into groups B and C; outpatient in a dedicated room in a rehabilitation center in Prague supported by REGIBASE Prague, where were enrolled in group A. The duration of the rehabilitation programs was 10 weeks in total. For eligible subjects in group A, MRI was performed twice: once during the weekend prior and once after the therapeutic program.

**Fig. 1:**
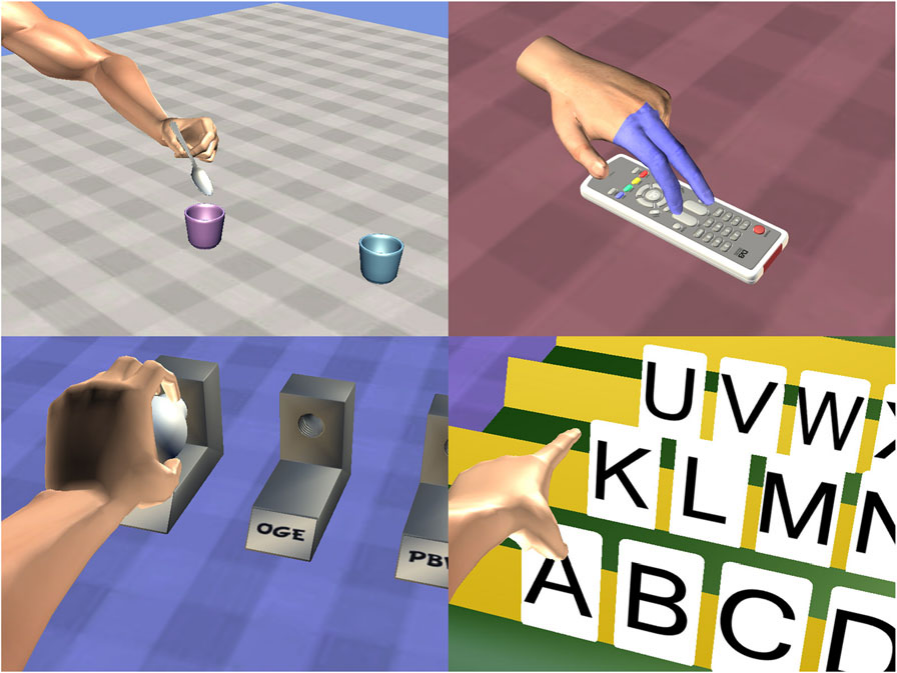
Examples of VAI games: multi-finger actions to pick up a spoon and drop it into a cup, tapping actions using the index and middle fingers on a remote control, removing a light bulb and reinserting it into another fixture designated by a letter of the alphabet, choosing letters of the alphabet to form words and phrases. All actions are performed by clicking and draging mouse on the appropriate body part

VAI therapy included several exercises and tasks. In all tasks, the subjects used a standard personal computer with a mouse, which was controlled by the unimpaired upper extremity. Using the mouse, subjects moved the upper extremity on the computer screen, which was an image of the corresponding impaired limb (i.e., a subject with right-sided paresis controlled the mouse with their healthy left hand and moved the virtual impaired right arm or hand on the screen). The following exercises are embedded in VAI gameplay for simulating tasks encountered in activities performed during daily living, namely hand exercises included virtual: 1) pincer actions to grasp a key, 2) two-finger actions to grasp a ball and drop it into a cup, 3) multi-finger actions to pick up a spoon and drop it into a cup, 4) full-hand grasps around a mug handle, 5) tapping actions using the index and middle fingers on a remote control, 6) hand grasps of objects shaped as stars, circles, or squares, followed by placing the objects in similarly shaped slots., Some of the tasks simulated occupational therapy: 7) upon request, opening a particular numbered box from a display of many numbered boxes; 8) removing a light bulb and reinserting it into another fixture designated by a letter of the alphabet; 9) moving multiple jigsaw puzzle pieces into place; 10) selecting numbers by choosing a desired form of calculation and making a computation; and 11) choosing letters of the alphabet to form words and phrases (see Fig. 1). All actions are performed by selecting an anatomical body part by left clicking on the selection, holding the left click button down while dragging the selection to simulate an unimpaired physical movement.

P/OT consisted of 60 min of therapy for upper extremity activities for daily living training, 60 min of physical therapy for upper/lower/trunk therapeutic exercises, some group therapy, aquatic therapy, and group exercises. Full P/OT was performed every workday (5 days per week).

The subjects of Group A received VAI therapy only and averaged approximately 30 min per session, three times weekly, i.e., an average of 1.5 h per week (an average of 15 h intervention time in total). Group B subjects received, on average, the same duration of VAI therapy (i.e., 15 h) and 15 h of P/OT each week. Group C subjects received, on average, 15 h of P/OT each week, without any VAI intervention.

MRI was performed on 10 subjects in group A (*n* = 10; four subjects were unable to undergo MRI: three due to metal implants and one due to claustrophobia), using a 3 T Skyra scanner (Siemens, Erlangen, DE) equipped with a 32-channel head coil. Subjects from groups B and C were not scanned for logistical reasons (the distance between the rehabilitation and MR facility, the transport duration and costs). Structural images were obtained using a three-dimensional T1-weighted magnetization-prepared gradient-echo sequence (MPRAGE) (repetition time = 2500 ms; echo time = 4.82 ms; TI = 1100 ms; acquisition matrix = 256 × 256 × 192; flip angle = 7°; voxel size = 1 mm isotropic) and processed in VBM8 toolbox, (http://dbm.neuro.uni-jena.de/vbm.html) and the SPM8 software package (http://www.fil.ion.ucl.ac.uk/spm) using default parameters. As the first step, the brains of subjects with use of their right hand (with majority of symptoms on the left side) were flipped to achieve homogeneous laterality of training-related changes. The VBM8 toolbox involves bias correction, tissue classification, and affine registration. The affine registered grey matter (GM) and white matter segmentations were used to build a customized DARTEL template (diffeomorphic anatomical registration through exponentiated Lie algebra), creating warped GM and white matter segments. Modulation was applied in order to preserve the volume of a particular tissue within a voxel by multiplying voxel values in the segmented images by the Jacobian determinants derived from the spatial normalization step. In effect, the analysis of the modulated data tests for regional differences in the absolute amount (volume) of GM. Finally, images were smoothed with a FWHM kernel of 8 mm. Statistical analysis was carried out by means of a whole-brain paired t-test between pre- and posttest. The threshold of the results was *p* < 0.005 nonstationary smoothness and cluster corrected. For the purpose of ROI analyses the mean pre- and posttest grey matter volumes were extracted using the Marsbar toolbox and then the difference values were used for the correlation. Mean grey matter volume changes in the significant clusters were correlated with active range of motion (AROM) and grip power. The threshold of the results was p < 0.005 nonstationary smoothness and cluster corrected.

Physical power measurements included a pinch gauge (MG-4320NC pinch gauge: B and L) to measure key pinch and three-jaw chuck strength for the impaired extremity. Pinch strength was measured in kilograms of pressure. A Jaymar dynamometer was used to measure the gross grasp and power grip of the impaired extremity. Grasps were measured in kilograms of pressure.

The upper extremity’s impaired-side active range of motion was measured by goniometric measurements for shoulder abduction, flexion, elbow flexion, and wrist extension.

The Action Research Arm Test (ARAT) measured the ability to perform gross movements and the ability to grasp, move, and release objects that differed in weight, shape, and size [20]. No formal assessment of hemispatial neglect was performed.

The spasticity of all muscle groups of the upper extremity was measured on the Modified Ashworth Scale [21, 22]. All clinical scores were assessed by a single trained and certified experienced physiotherapist (JP).

The Statistical Package for Social Sciences (SPSS version 22) was used for data analysis, and charts comparing group differences were plotted using LibreOffice 5.2.7.2 and Inkscape 0.92. A descriptive analysis was completed to explore group differences in age and elapsed time from injury, using one-way analysis of variance (ANOVA). A mixed-model repeated measures ANOVA was employed to compare differences in groups from the preintervention baseline to postintervention change. As the VAI group (A) received only about 15 h of intervention total on average compared to the 15 h per week that groups B and C received within the timeframe of the study (see Table 1), we introduced therapy hours as a covariate and conducted one-way analysis of covariance (ANCOVA, *p* < 0.10). Also, since the groups were not homogeneous at the baseline, baseline scores for the corresponding variables were also controlled and adjusted mean scores were computed. Because baseline scores were considerably lower (subjects were more severely impaired) for VAI intervention across all variables; adjusted mean scores were compared to depict the motor gains from the baseline to postintervention.

A one-tailed Pearson correlation analysis (*p* < 0.05) was used to verify relationships between the gains recorded in motor variables on the affected side of the body and MRI-measured changes in grey matter volumes in key regions of the brain for 10 subjects in group A before and after VAI intervention.

The primary outcome of the study were the changes (between 1 and 3 days before intervention and 10-weeks post-intervention) in cortical grey matter volume (also correlated with quantified motor skills) and changes in the motor skills (muscle strength and AROM), there was no secondary outcome measure.

## Results

ANOVA did not reveal group differences in terms of age, but they were different in terms of time postinjury (df = 2, F = 7.6, *p* = 0.002), where subjects in group A were significantly longer after ABI (average 6.9 years postinjury). The groups were also significantly different in terms of average spasticity: measured on the Modified Ashworth Scale (df = 2, F = 3.3, *p* = 0.04) the three groups (A, B, and C) scored 2 (SD = 1.4), 1 (SD = 1.08), and 0.75 (SD = 1.16), respectively (Tables 1, 2, 3 and 4).

**Table 3:** Action Research Arm Test (ARAT) changes in group A. ID = group and patient ID, IE = impaired upper extremity, L = left, R = right, pre = baseline before VAI intervention, post = after VAI intervention

ID	IE	ARAT L pre	ARAT L post	ARAT R pre	ARAT R post	Affected side ARAT change	Unaffected side ARAT change
A01	L	5	52	57	57	47	0
A02	L	3	3	57	57	0	0
A04	R	57	57	3	3	0	0
A05	R	56	57	57	57	0	1
A06	R	57	57	52	57	5	0
A07	L	54	56	57	57	2	0
A08	R	57	57	5	57	52	0
A09	R	54	55	57	57	0	1
A10	R	57	57	2	2	0	0
A11	L	5	52	57	57	47	0
A12	R	57	57	12	13	1	0
A13	L	4	41	57	57	37	0
A14	L	45	56	57	57	11	0
A15	L	41	53	41	47	12	6
B01	L	57	57	57	57	0	0
B03	R	57	57	41	53	12	0
B04	R	57	57	36	57	21	0
B06	R	57	57	56	56	0	0
B07	L	16	22	57	57	6	0
B10	L	0	0	57	57	0	0
B12	R	57	57	0	2	2	0
B15	L	53	57	53	57	4	4
B17	L	33	48	57	57	15	0
B19	R	57	57	3	23	20	0
B20	R	56	57	0	0	0	1
B21	R	24	53	57	57	29	0
B23	R	57	57	56	56	0	0
C01	R	40	40	40	40	0	0
C02	L	57	57	57	57	0	0
C03	R	57	57	0	10	10	0
C04	R	57	57	30	35	5	0
C05	R	57	57	52	57	5	0
C07	L	8	41	56	57	33	1
C11	L	54	53	56	57	-1	1
C12	L	55	55	55	55	0	0

**Table 4:** Correlation between strength and shoulder motion active range of motion, and grey matter volume (BA = Broadmann area, N.S. = not significant, CL = contralesional; values without CL are ipsilesional – on the side of the brain lesion)

Brain region	Grasp strength	Shoulder abduction	Shoulder flexion	Elbow flexion
Precentral gyrus	r = 0.6 / p = 0.04	N.S.	N.S.	r = 0.6 / *p* = 0.03 CL
Supplementary motor area	R = −0.6 / p = 0.03	r = 0.65 / *p* = 0.02 r = 0.8 / *p* = 0.003 CL	r = 0.8 / p = 0.003 r = 0.8 / *p* = 0.004 CL	r = −0.6 / p = 0.03
BA 6	N.S.	r = 0.6 / p = 0.03	r = 0.6 / p = 0.03	N.S.

### Strength and range of motion

**Fig. 2:**
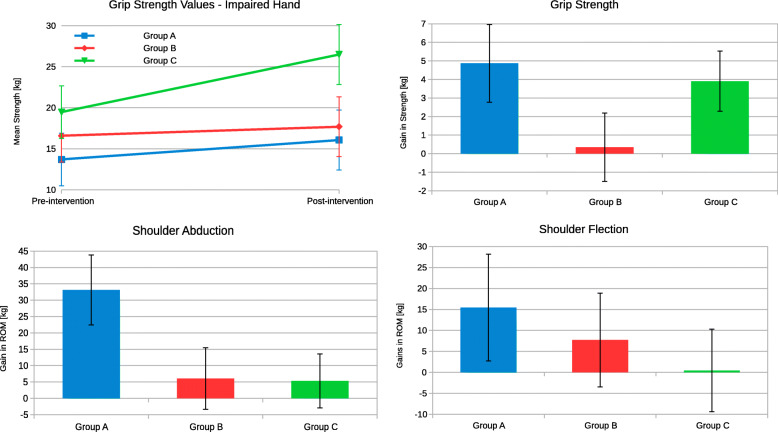
Motor achievements: **a** grip strengths before and after intervention. In parts **b**–**d**, estimated marginal means of gains are presented for **b** grip strengths, **c** shoulder abduction, and **d** shoulder flexion. In graphs **b**, **c**, and **d** the covariates appearing in the model are evaluated at the following values: therapy hours = 152.2466

All participants demonstrated improvements in the affected upper extremity in at least one of the following measures: hand-key pinch strength, grasp, and three-jaw chuck pinch, shoulder AROM, elbow AROM, wrist AROM, and shoulder flexion AROM. Key pinch strength improved after therapy in groups A and B. After controlling for therapy hours, a significant difference between the groups was observed (F(2, 34) = 2.5, *p* = 0.09), with group A demonstrating the highest improvement in key pinch strength. Without controlling for therapy hours, the three-jaw chuck pinch improved between pre- and postintervention only in groups B and C. Again, after controlling for therapy hours, a significant difference was present (F(2, 34) = 5.1, *p* = 0.01) and group A showed the highest gains. Gross and power grips improved in the three groups in various extents (Fig. 2a). When the therapy hours were controlled for strength, improvements in power grasps and grips were higher for group A (F(2, 34) = 1.7, *p* = 0.19, Fig. 2b).

AROM in the shoulder was measured for abduction and flexion, with all three groups making improvements, though the differences were not statistically significant. After correcting gains in shoulder abduction for therapy hours, improvement in group A (33°) was even more pronounced than in groups B and C (Fig. 2c). The difference did not reach the statistical threshold due to the high deviations in AROM (Fig. 2c and d).

Elbow flexion increased the most in group A (14°), compared to groups B and C, though no statistical difference was detected on the mixed-model ANOVA. After controlling for therapy hours, the highest gains remained in group A; however, the difference was not statistically significant across the three groups on the ANCOVA (F (2, 34) = 0.48, *p* = 0.62). Finally, the same scenario was noted in wrist extension, where improvements were most noticeable in group B (11°), followed by group A (9°). No statistical difference was detected between the groups.

The three groups demonstrated modest improvements in upper extremity movement with ARAT (Table 3), predominantly in subjects with left upper limb impairment – of 5 subjects, who increased their ARAT score by more than 30 points after the rehabilitation,four had left and only one had right upper limb impairments. Four of five subjects with high ARAT improvement were in group A.

With therapy hours controlled for and with adjusted mean scores, subjects using only VAI (group A) improved the most. The difference, however, was not statistically significant (F (2,-34) = 0.4, *p* = 0.5).

### Voxel-based morphometry of MRI

**Fig. 3:**
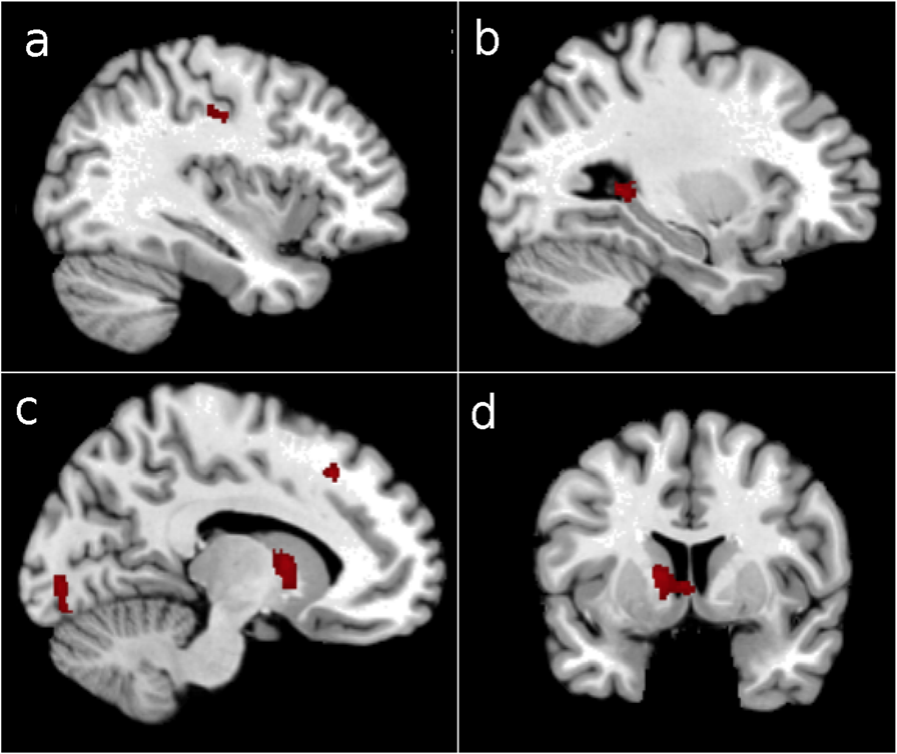
Results of voxel-based morphometry, nonstationary smoothness corrected *p* < 0.005, cluster size > 200. Neurological convention (left side of the brain is on the left side of the picture). Grey matter (GM) volume increased in the left postcentral gyrus (**a**), tail of the left hippocampus (**b**), left visual cortex and left prefrontal areas 8 and 32 (**c**), and left caudate (**d**)

In the 10 subjects that used only VAI (part of group A), voxel-based morphometry, based on morphological T1-weighted MRI data, proved volumetric grey matter increases in five brain areas (aside from traditional VBM output in Fig. 3, we also calculated AAL atlas-based changes in gray matter probability — the given numbers are the mean/median increase in probability): the left hippocampal tail (10.91%/11.44%), left caudate nucleus (8.96%/7.40%), left rostral cingulate zone (4.13%/4.08%), depth of left central sulcus (6.30%/5.81%), and left visual cortex (7.42%/7.89%). All subjects in this subgroup demonstrated positive functional motor changes on the impaired side as measured by key pinch and three-jaw chuck pinch strength, shoulder abduction AROM, elbow AROM, wrist AROM, and shoulder flexion AROM.

Several significant correlations between gains in motor scores, active ranges of motion, and grey matter volumes were detected (for correlation coefficients and *p*-values, see Table 4).

Five significant correlations (high or very high strength) between gains in motor variables of impaired extremities and volumetric changes in the impaired grey matter were detected: grasp strength and the impaired precentral gyrus, gains in shoulder abduction AROM and the impaired supplementary motor area, gains in shoulder abduction AROM and the impaired Broadmann area 6, gains in AROM for shoulder flexion and the impaired supplementary motor area, and gains in AROM for shoulder flexion and the impaired Broadmann area 6.

Three significant correlations were also detected between gains in motor measures and volumetric grey matter changes in unimpaired regions of the brain. These correlations were between AROM for elbow flexion and the precentral gyrus, AROM for shoulder abduction and the unimpaired supplementary motor area, and AROM for shoulder flexion and the unimpaired supplementary motor area.

Two inverse correlations of significance were also detected: grasp strength gains and the impaired supplementary motor area, and AROM gains in elbow flexion and the impaired supplementary motor area.

## Discussion

The main findings of the present study are that including VAI exercises and tasks in the rehabilitation program significantly improves the motor function and skills of impaired upper extremities in subjects with ABIs. This was proven by the different subjective and objective functional measurements and scales, e.g. ARAT, AROM, key pinch, gross power, and grasp, as well as documented by a significant increase in grey matter volume in the motor and premotor regions of the brain.

VAI intervention is one of a growing number of VW rehabilitation programs specifically designed to restore movement to paretic upper extremities [19], which provide anatomically realistic virtual upper extremities to simulate intended movements of an impaired extremity. In previous studies, the positive effects of VAI on the improvement of motor functions in upper extremities has already been reported [19, 23–26] and is related to the findings of Kühn et al. [13, 14]. An earlier trial with adult survivors of ABI using VAI [19] resulted in clinically significant improvements in range of motion and strength, clinical improvements in active movements for shoulder and wrist flexion, and clinically relevant improvements in elbow flexion and upper extremity strength.

Our study supports this observation based on significantly improved upper extremity function after VAI seen in both the muscle strength and active range of motion, especially in the key pinch, three-jaw chuck, and power grip. The subjects of group A (who received VAI only) were in the more chronic stage of ABI, with longer elapsed average time postinjury and a higher level of spasticity. They underwent fewer hours of rehabilitation (and no P/OT during VAI intervention), even though they presented higher motor improvement than seen in comparison to the other groups B and C. Having the same extent of computer rehabilitation and P/OT would be beneficial from the statistical perspective, but spending 15 h of physical rehabilitation and 15 h with a computer per week (which means 6 h daily during workdays) may be for some of the participants too much (especially for those who are not used to exercise in their home environment). Moreover, the design of the VAI training would have to be modified for shorter training blocks three times per day. In group B, one measure (grasp strength) declined. It is unclear how much VAI intervention or P/OT might account for the change in the outcome of this variable for group B. Since P/OT therapy hours for group B participants were in addition to the VAI-only therapy undergone by group A, we cannot attribute the declines in grasp strength to the use of VAI. One possible explanation is that group B participants experienced physical and occupational therapy fatigue or another effect of ‘override’ or interference, contributing to decline in strength. In conjunction with the motor skill improvements noted for group A, improvements are present in functional measures, namely the ARAT (Table 3), which has been used in recent meta-analytic study [27] as one of the major outcome measures to assess the effectiveness of virtual reality interventions for upper extremity rehabilitation post-stroke. Even though our results did not reach statistical significance due to small group sizes, they reveal a clear pattern: only one of the subjects in this group (A02) with left-sided impairment did not improve in ARAT (and 6 improved). On contrary, only two of right-sided subjects improved (A08 by 52 points, A06 5 points) but 5 did not improve at all. Along with the fact, that the high improvement in ARAT (four of 14 subjects of group A) was not observed in group B suggests, that isolated VAI therapy may be beneficial especially for subjects with right-sided impairment.

As in strength measurements, AROM measurements with therapy hours controlled for demonstrated substantive improvements for group A that were on par or better than found in groups B and C. In group B (P/OT and VAI), similar results were noted as for those in group A (VAI only). Improvements in shoulder AROM were not statistically significant; however, after correcting for therapy hours, group A had higher gains than the other groups. It is worth mentioning that in group A, four individuals with left-sided impairment improved highly in AROM and only one did not improve at all; this is contrary to subjects with right-sided impairment, where a half did not improve in AROM.

Wrist extension and elbow flexion on the impaired side increased most in group A, and the difference was statistically significant.

### Voxel-based morphometry of MRI

Even though VBM is well-established method and its stability and reliability has been published [28], the underlying cellular events in gray matter regions (including axonal sprouting, dendritic branching and synaptogenesis, neurogenesis, changes in glial number and morphology, and angiogenesis) and possible changes in the white matter (alterations in fiber organization, axonal branching, sprouting, changes in packing density, axon diameter, fiber crossing and the number of axons, myelination of previously unmyelinated axons, changes in myelin thickness and morphology, changes in astrocyte morphology or number or even angiogenesis) were suggested [29], but not confirmed on the microscopic basis.

VR and, especially, VW research has also observed functional improvements associated with structural brain changes measured by MRI, including activation of the supplementary motor area, premotor cortex, and the primary motor cortex during VR training [30]. Activations have been observed in areas of the brain involved in motor preparation, including the anterior intraparietal area and the supplementary motor area.

In our study, the cortical volume of both subcortical and cortical regions increased after VAI. In the subcortical structures, we found an increase in GM volume in the hippocampus, rostral cingulum, and caudatum. These structures are involved in the formation of the processes of learning and emotions. Recorded benefits include maximizing neuroplastic processes via motor learning and motor recovery [24]. Specific motor representations associated with motor imagery involve parietofrontal circuitry and the activation of the contralesional motor areas, inferior and middle temporal gyrus, and ipsilesional anterior lobe of the cerebellum [31, 32].

After VAI, we observed a GM volume increase in the depth of central sulcus, which is in agreement with exercise studies published on, for example, elderly subjects after a dance training program [33]. We also found an increase of GM volume in the visual cortex, which can be attributed to the stimulation by a visual representation of the limbs on the screen [34].

After VAI, AROM increased and correlations of these improvements were found in cortical GM volume. In VR studies of stroke-affected adults, results demonstrated reorganization and increased activation in the ipsilesional motor cortex [35]. Our study supports this with a correlation between changes in Brodmann area 6, shoulder flexion, and abduction AROM. Moreover, Orihuela-Espina and associates found contralesional activation of the unaffected motor cortex, cerebellar recruitment, and compensatory prefrontal cortex activation were the most prominent changes noted in fMRI studies of ABI-affected participants using VR technologies [25]. This is in agreement with our findings of positive correlations of SMA with shoulder flexion and abduction AROM on both impaired and unimpaired sides as well as a positive correlation of the precentral gyrus and elbow flexion AROM on the unimpaired side.

Our results relate to previously published observations that the premotor cortex is responsible for motor initiation and motor-control coding for skilled motor sequences, and the internal generation of mental imagery of the task or motor movement [19, 30–32, 35]. In the present study, the amount of probable volumetric changes in GM in the precentral gyrus for grasp and key pinch improvements resulting from VAI intervention, together with positive correlations of shoulder flexion and abduction AROM with SMA and Brodmann area 7, imply neocortical reorganization. VAI is supposed to activate parts of the brain responsible for imagining, planning, and performing physical tasks in damaged areas. Sampson and Shau found similar results, with participants experiencing increased shoulder and elbow strength [26]. Kwon and Park, in comparing two groups, one receiving VW rehabilitation and the other a control group, found the VW group showed significant improvement in upper extremity function related to manual dexterity and basic activities performed during daily living [23]. For both grasp and elbow flexion strength, an inverse relationship was found: as strength improved, volumetric GM in the supplementary motor area (as measured by MRI) decreased. This relationship may reflect the responsibility of the supplementary motor area in planning complex movements with high integration of visual information. It appears that as basic grasp and elbow flexion strength improve and become more routine, the planning and need for visual input for these movements decreases, leading to a decrease in cortical thickness.

### Future plans

A future study with a larger sample size and random assignment of subjects ensuring homogeneity (in terms of time since injury, baseline scores, and therapy time) is needed to corroborate the above findings. Future research should include neuroimaging to continue to clarify what is occurring at the brain’s structural level, which has already been reported by our study group using other rehabilitation techniques such as motor program activating therapy [10].

### Study limitations

The purpose of this study was to examine the feasibility and preliminary effectiveness of integrating VR exercise interventions in a full-fledged rehabilitation setting. Due to logistical barriers to recruitment, including overall cost and proximity to MRI facilities, survivor-participant groups were not fully randomized (the randomization was performed between groups B and C, for details see the methodology section). Due to ethical considerations, participants had to be enrolled on a self-selected (volunteer) basis at each site without altering their existing therapy regimen. As a result, group homogeneity was not possible. For example, group A participants were in the chronic stages of recovery and had more severe levels of spasticity. Due to the group inhomogeneity, the ARAT scores also had relatively higher variability. The small sample size restricts the generalization of these findings to the broader ABI population without further study.

## Conclusion

The present study supports the existing literature, which finds that ABI survivors can make functional improvements months after the onset of injury. The results of this study support the existing literature in reporting changes at the structural level through neural growth, at the activity level through improvements in different measures of muscular strength and ranges, and at the participation level through engagement with the VAI software and games. These findings demonstrate the feasibility of incorporating VAI therapy in multiple settings including acute, sub-acute, and outpatient rehabilitation services. Even with the age, time, and postevent disadvantages of groups A and B compared to group C, the results suggest there is value in VAI intervention, specifically in regaining structural and functional improvements before disabled extremities can be autonomously moved. Volumetric changes in the GM of motor and premotor areas resulting from VAI intervention imply neocortical reorganization due to structural brain plasticity changes responsible for imagining, planning, and performing physical tasks in the damaged areas of the brain.

The clinical relevance of these findings for rehabilitation professionals is twofold; first they provide a broader choice of modalities for treatment, which can influence recovery, and second they provide patients with an avenue for creating an independent method of continuing the recovery process beyond their time with a rehabilitation professional. This second point is critical, as the ability for recovery through focused practice and skill training extends well beyond the timeframe of working with a rehabilitation professional and may improve outcomes associated with pinch, grasp, and AROM. Although this study did not include measurements of function, it is hypothesized that with the increased function of the upper extremity, subjects will attempt to use the extremity more in daily tasks. This hypothesis is further supported by the improved AROM in the shoulder and elbow of the involved extremity; this range of improvement could allow for object manipulation and activity participation with bilateral upper extremities as opposed to unilateral effort and activities limited to tabletops or supported work against gravity.


**Additional file 1: Video 1.** Demonstration of the use of virtual limb – subject controls the virtual limb on the screen with mouse and than tries to accomplish the same task with his real contralateral (in this case left) limb.

## Data Availability

The datasets during and/or analyzed during this study are available from the corresponding author on reasonable request.

## Abbreviations

ABI: Acquired brain injury
ANOVA: One-way analysis of variance
ANCOVA: One-way analysis of covariance
ARAT: Action Research Arm Test
AROM: Active range of motion
BA: Brodmann area
CL: Contralesional
CVA: Cerebrovascular accident
GM: Grey matter
MRI: Magnetic resonance imaging
P/OT: Physical/occupational therapy
TBI: Traumatic brain injury
VAI: Virtual anatomical interactivity
VBM: Voxel-based morphometry
VR: Virtual reality
VW: Virtual world
